# Terfenadine, a histamine H1 receptor antagonist, induces apoptosis by suppressing STAT3 signaling in human colorectal cancer HCT116 cells

**DOI:** 10.3389/fphar.2024.1418266

**Published:** 2024-06-13

**Authors:** Manoj Kumar Baniya, Eun-Hee Kim, Kyung-Soo Chun

**Affiliations:** ^1^ College of Pharmacy, Keimyung University, Daegu, Republic of Korea; ^2^ College of Pharmacy and Institute of Pharmaceutical Sciences, CHA University, Seongnam, Republic of Korea

**Keywords:** terfenadine, histamine H1 receptor, apoptosis, STAT3, colorectal cancer

## Abstract

**Introduction:**

Colorectal cancer is a highly aggressive and metastatic cancer with inadequate clinical outcomes. Given the crucial role of histamine and histamine receptors in colorectal carcinogenesis, this study aimed at exploring the anticancer effects of terfenadine against colorectal cancer HCT116 cells and elucidate its underlying mechanism.

**Methods:**

Herein, we examined the effect of terfenadine on growth and proliferation of HCT116 cells *in vitro* and *in vivo*. Various experimental techniques such as flow cytometry, western blot, immunoprecipitation, luciferase assay were employed to unveil the mechanism of cell death triggered by terfenadine.

**Results:**

Terfenadine markedly attenuated the viability of HCT116 cells by abrogating histamine H1 receptor (H1R) signaling. In addition, terfenadine modulated the balance of Bax and Bcl-2, triggering cytochrome c discharge in the cytoplasm, thereby stimulating the caspase cascade and poly-(ADP-ribose) polymerase (PARP) degradation. Moreover, terfenadine suppressed murine double minute-2 (Mdm2) expression, whereas p53 expression increased. Terfenadine suppressed STAT3 phosphorylation and expression of its gene products by inhibiting MEK/ERK and JAK2 activation in HCT116 cells. Furthermore, treatment with U0126, a MEK inhibitor, and AG490, a JAK2 inhibitor, dramatically diminished the phosphorylations of ERK1/2 and JAK2, respectively, leading to STAT3 downregulation. Likewise, terfenadine diminished the complex formation of MEK1/2 with β-arrestin 2. In addition, terfenadine dwindled the phosphorylation of PKC substrates. Terfenadine administration (10 mg/kg) substantially retarded the growth of HCT116 tumor xenografts *in vivo*.

**Conclusion:**

Terfenadine induces the apoptosis of HCT116 cells by abrogating STAT3 signaling. Overall, this study supports terfenadine as a prominent anticancer therapy for colorectal cancer.

## 1 Introduction

Colorectal cancer (CRC) line up third in terms of incidence and prevalence, with an incidence rate of 10.2% worldwide. The survival rate of metastasizing CRC is <20%. However, the results of the latest 5-year clinical trials revealed that the overall survival rate is increased with marked improvement in the pathophysiological characteristics of the tumor (Biller and Schrag, 2021). Various conventional treatments are available for CRC, depending on the disease condition. Different types of combination therapies, including immunotherapy and chemotherapy, are being developed by researchers to overcome CRC-associated multidrug resistance (Dariya et al., 2020). Although various immunotherapies and targeted therapies are available, CRC exhibiting metastatic characteristics poses a great challenge in its therapeutic management (Messersmith, 2019). Interestingly, the development of new chemotherapeutic agents with better therapeutic activity can prove to be a boon in the successful treatment of CRC.

Apoptosis is a programed cell death modality involving a caspase cascade through intrinsic and extrinsic mechanisms and their downstream targets (Kim and Choi, 2013; Kashyap et al., 2021; Morana et al., 2022). Cancer cells tend to bypass apoptotic cell demise by distorting the homeostasis between antiapoptotic B-cell lymphoma (Bcl) family proteins and proapoptotic proteins (Hanahan and Weinberg, 2011; Kundu et al., 2014). An imbalance between proteins that regulate apoptosis is a major factor in many cancers (Elmore, 2007; Park et al., 2014; Yun et al., 2017). Alterations in the proportions of Bax and Bcl-2 destabilize the membrane potential of the mitochondria, leading to the transfer of cytochrome c into the cytoplasm, which triggers the simultaneous cleavage of caspase-9, caspase-7, and caspase-3 and PARP inducing cell death (Kim, 2014; Chae et al., 2020). The expression of Bcl-2 is modulated by the signal transducer and activator of transcription-3 (STAT3) at the transcriptional level (Kundu et al., 2014). STAT3 activation is triggered through phosphorylation by several upstream regulators such as MEK/ERK (Aggarwal et al., 2009) and JAK2 (Slattery et al., 2013; Kim et al., 2016), followed by dimer creation and transfer into the nucleus for transcriptional activity. Transient STAT3 activation promotes normal cell growth and differentiation, whereas constitutive activation is associated with carcinogenesis, apoptosis evasion, and cellular proliferation. The downregulation of STAT3 activation suppresses cellular proliferation and prompts apoptosis (Aggarwal et al., 2009; Johnston and Grandis, 2011; Cho et al., 2014; Park et al., 2019; Raut et al., 2021).

Histamine H1 receptor (H1R) is a Gαq/11-coupled transmembrane receptor (Parsons and Ganellin, 2006; Lieberman, 2011). The activation of this receptor activates phospholipase C, which mediates the stimulation of protein kinase C (PKC) and discharge of calcium via the assembly of inositol triphosphate (IP3) and diacylglycerol (DAG), which function as second messengers for downstream signal transduction. PKC exerts a significant role in the G protein-associated signaling transducer mechanism of H1R through the phosphorylation of several substrates mediating downstream signaling (Massari et al., 2020; Nguyen and Cho, 2021). It is reported to be regulatory upstream of ERK, which is a mitogen-activated protein kinase (MAPK) and critical player in carcinogenesis (Matsubara et al., 2005). However, the complex formation of G protein-coupled receptors (GPCRs) with β-arrestins can actuate G protein-independent signaling mechanisms (Luttrell and Luttrell, 2004; Park et al., 2016; Moo et al., 2021). Arrestins represent scaffold proteins that pairs with G protein-coupled receptor kinase (GRK)-mediated phosphorylated GPCRs, causing the desensitization of the receptors and reduction of their response to the ligand. In addition to the desensitization as scaffold functions of β-arrestins, particularly β-arrestins 1 and 2, they recruit several signaling proteins, including components of MAP kinase cascades and Src family tyrosine kinases, and act as signal transducers irrespective of G protein activation (Miller and Lefkowitz, 2001; Perry and Lefkowitz, 2002; Chun et al., 2010).

Numerous studies have suggested that histamine is a critical player in colorectal carcinogenesis. CRC cells display aberrant H1R expression, implying the tumorigenic function of histamine (Wang et al., 2014; Massari et al., 2020). In addition to the predominant expression of H1R, CRC cells demonstrate expression of other types of histamine receptors including H2R and H4R. Even the mRNA levels of these histamine receptors were reported to be higher in CRC than that of normal mucosa (Cianchi et al., 2005). Conversely, another study showed reduced expression of H1R and H4R in CRC as compared to normal mucosa (Boer et al., 2008). Microarray studies reflect high expression of H1R positively correlated with poor survival of CRC cases (Wang et al., 2014). A preclinical study reported that exogenously administered histamine accelerates the proliferation of *in vivo* tumor xenografts in mice, corroborating the link between histamine and CRC (Tomita and Okabe, 2005). Clinical studies have also indicated a positive crosstalk between histamine receptors and CRC progression. (Chanda and Ganguly, 1987; Reynolds et al., 1997). Based on these findings, we speculated that histamine receptor antagonists could exert prominent anticancer effects on CRC.

Terfenadine is a second-generation antihistamine initially exploited for the management of allergic rhinitis and urticaria (McTavish et al., 1990). The clinical use of terfenadine was associated with several side effects, such as torsades de pointes and ventricular fibrillation, which caused its withdrawal from the market (MacConnell and Stanners, 1991; Delgado-Ramírez et al., 2021). However, recent studies have dissected the new interface of terfenadine, particularly its antitumor property, which could support its revival for therapeutic use. The idea of repositioning terfenadine as an anticancer agent has garnered considerable interest in the research community for exploration of its anticancer potential (Delgado-Ramírez et al., 2021). Although a few studies have delineated the antitumor property of terfenadine, its role against CRC and its underlying mechanism remain unexplored.

In this study, we explored the effect of terfenadine on the growth and proliferation of CRC HCT116 cells *in vitro* and *in vivo* by elucidating the mechanism of its anticancer properties. The impact of terfenadine on the stimulation of apoptosis and H1R-dependent signaling cascades in HCT116 cells was also inquired.

## 2 Materials and methods

### 2.1 Chemicals and reagents

Terfenadine, ranitidine, thioperamide, JNJ7777120, and AG490 were procured from Cayman Chemical Co. (Ann Arbor, MI, United States). Histamine, hydroxyzine, and 2-(2-pyridyl)ethylamine were obtained from Sigma-Aldrich (St. Louis, MO, United States). U0126, antibodies to cleaved caspase-9, caspase-3, caspase-7, PARP, Bcl-2, Bax, cytochrome c, ERK1/2, p-ERK1/2, STAT3, p-STAT3 (Tyr705), MEK1/2, p-STAT3 (Ser727), JAK2, p-JAK2, p-MEK1/2, cyclin D1, p-PKC, and secondary antibodies were procured from Cell Signaling Technology Inc. (Beverly, MA, United States). Anti-survivin antibody was bought from Novus Biologicals (Littleton, CO, United States). Antibodies against Mdm2, p53, β-arrestin 2, cyclin D2, and cyclin D3 were obtained from Santa Cruz Biotechnology (Dallas, TX, United States). Antibodies against H1R, H2R, and H3R were bought from Abcam (Cambridge, United Kingdom). Antibodies against H4R were secured from Alpha Diagnostic International (San Antonio, TX, United States). [3-(4,5-dimethylthiazol-2-yl)-5-(3-carboxymethoxyphenyl)-2-(4-sulfophenyl)-2H-tetrazolium] (MTS) and Fugene HD transfection reagent were obtained from Promega (Madison, WI, United States). The bicinchoninic acid protein assay kit was procured from Pierce Biotechnology (Rockford, IL, United States). The FITC-Annexin V staining kit was obtained from BD Biosciences (San Jose, CA, United States). Super signal WesternBright™ ECL HRP Substrate was acquired from Advansta (San Jose, CA, United States). Pierce Protein G Agarose and Supersignal™ West Femto Maximum Sensitivity Substrate were procured from Thermo Scientific (Rockford, IL, United States). Ro31-8220 was obtained from APExBIo Technology (Houston, TX, United States).

### 2.2 Cell culture

Human CRC HCT116 cells were procured from the Korean Cell Bank (Seoul, South Korea). The complete media for the growth of HCT116 cells consisted of Dulbecco’s Modified Eagle Medium, heat-inactivated fetal bovine serum (10% v/v), and penicillin–streptomycin (1% v/v). The cells were grown in a humidified atmosphere at 37°C with an adequate supply of 5% CO_2_.

### 2.3 Cell viability assay

Cell viability was assessed employing the MTS assay method previously described (Kundu et al., 2014). Briefly, 2 × 10^3^ cells were plated in 96-well plates. Cells were stimulated with different strengths of terfenadine in the presence or absence of histamine and 2-(2-pyridyl) ethylamine for the indicated time points in 100 µL of media. After treatment, the MTS reagent was mixed with the culture media in a ratio of 20:80. The cells were treated with the MTS solution for 1 h, and the optical density was recorded at a maximum wavelength of 490 nm using a Versamax microplate reader (Molecular Devices, San Jose, CA, United States).

### 2.4 Annexin V staining

Quantitative analysis of apoptosis was performed through flow cytometry utilizing a FITC-Annexin V staining kit. Annexin V staining was carried out as per the manufacturer’s guidelines. Briefly, cells were plated in a 6-well plate at a density of 2 × 10^5^ cells per well and incubated overnight. Then, terfenadine was administered to the cells at different doses for 24 h, as specified. Live and dead cells were collected and centrifuged. Binding buffer (100 µL) was added, and the cells were treated with Annexin V and propidium iodide (PI) for 15 min at room temperature. The fraction of terfenadine-induced apoptosis was quantified using a flow cytometer (BD Biosciences).

### 2.5 Western blot analysis

Briefly, cells were plated and stimulated with varying terfenadine strengths for 24 h. After collection, cell lysis was achieved using RIPA lysis solution followed by quantification of the collected proteins using a bicinchoninic acid protein assay kit. Moreover, 30–60 µg of protein samples were resolved on 8%–12% sodium dodecyl-sulfate polyacrylamide gel electrophoresis and then transferred onto polyvinylidene difluoride membranes. Skim milk solution in TBST (5% w/v) was used for membrane blocking by shaking the membranes at 75 rpm for 1 h. The membranes were probed with primary antibodies suitably diluted in TBST overnight at 4°C, followed by sequential washing with TBST and incubation with appropriate secondary antibodies (1:5000) at room temperature for 1 h. Super signal WesternBright™ ECL HRP Substrate or Supersignal™ West Femto Maximum Sensitivity Substrate was incubated with the membranes for 5 min and photographed using ImageQuant™ LAS4000 (Fujifilm Life Science, Tokyo, Japan) to obtain chemiluminescence images.

### 2.6 Luciferase reporter gene assay

Each well of six-well plates was plated with 4 × 10^5^ HCT116 cells and then placed in incubator overnight. Fugene HD transfection reagent was then used to transfect the cells with STAT3 and Renilla luciferase plasmids. The transfection process was performed following the manufacturer’s protocol. After 24 h of transfection, the cells were exposed to specified strengths of terfenadine, U0126, or AG490 for 24 h. Cellular protein was collected by treating the cells with passive lysis buffer. Finally, 20 µL of the obtained cell lysate was pipetted out into a 96-well plate, the luciferase assay reagent was added, and firefly luminescence was recorded using a microplate reader (Tecan, Mannedorf, Switzerland). Renilla luminescence was then recorded by adding Stop & Glo Reagent. Then, the firefly luminescence was divided with the Renilla luciferase reading, and the obtained values were plotted relative to the control.

### 2.7 Immunoprecipitation assay

Cells were exposed to the specified strengths of terfenadine for 24 h. Protein extraction was carried out using immunoprecipitation lysis solution, and the extracted lysate was incubated with Pierce Protein G Agarose in a rotary mixer for 1 h at 4°C. Thereafter, the protein was centrifuged, and the supernatant was collected. An appropriate primary antibody was added to 500 µg of protein and mixed overnight at 4°C in a rotary mixer maintained at 10 rpm. The immune complex formed was then separated by adding 30 µL of Pierce Protein G Agarose and incubated for 4 h. Thereafter, the agarose beads were separated by centrifugation and sequentially washed with immunoprecipitation lysis buffer five times. Denaturation buffer was added to the agarose beads and heated at 95°C for 10 min to release the protein of interest. Finally, the supernatant was collected by centrifugation and removal of agarose beads and used for further analysis.

### 2.8 *In vivo* tumor xenograft experiment

For the development of HCT116 tumor xenografts, 4- to 5-week-old BALB/c nude mice were used (Orient Bio, Inc., Seongnam, Korea). HCT116 cells (1 × 10^6^) were dispersed in a mixture of equal volumes of phosphate-buffered saline and matrigel and inoculated subcutaneously in the right flank area of the mice. After the tumor was grown to a size of approximately 100–150 mm^3^ following 7 days of cell implantation, three groups of mice were randomly created (*n* = 5 per group) as follows: control (corn oil), terfenadine 2 mg/kg, or terfenadine 10 mg/kg. The mice were administered corn oil or terfenadine daily through intraperitoneal injection for 20 days. The tumor volume and weight of mice were recorded twice a week during treatment. The tumor volume was determined using the equation: volume = (length × width^2^)/2. All murine experiments comply with the guidelines of the Animal Use Ethics Committee of Keimyung University.

### 2.9 Statistical analysis

Statistical analysis of data was carried out by ordinary one-way analysis of variance involving Tukey’s multicomparison test for intergroup comparison (GraphPad Prism Software, Boston, MA, United States). The results are presented as mean ± SEM (*n* = 3). A *p*-value of <0.05 was considered statistically significant.

## 3 Results

### 3.1 Effect of histamine receptor antagonists on HCT116 cell viability

**FIGURE 1:**
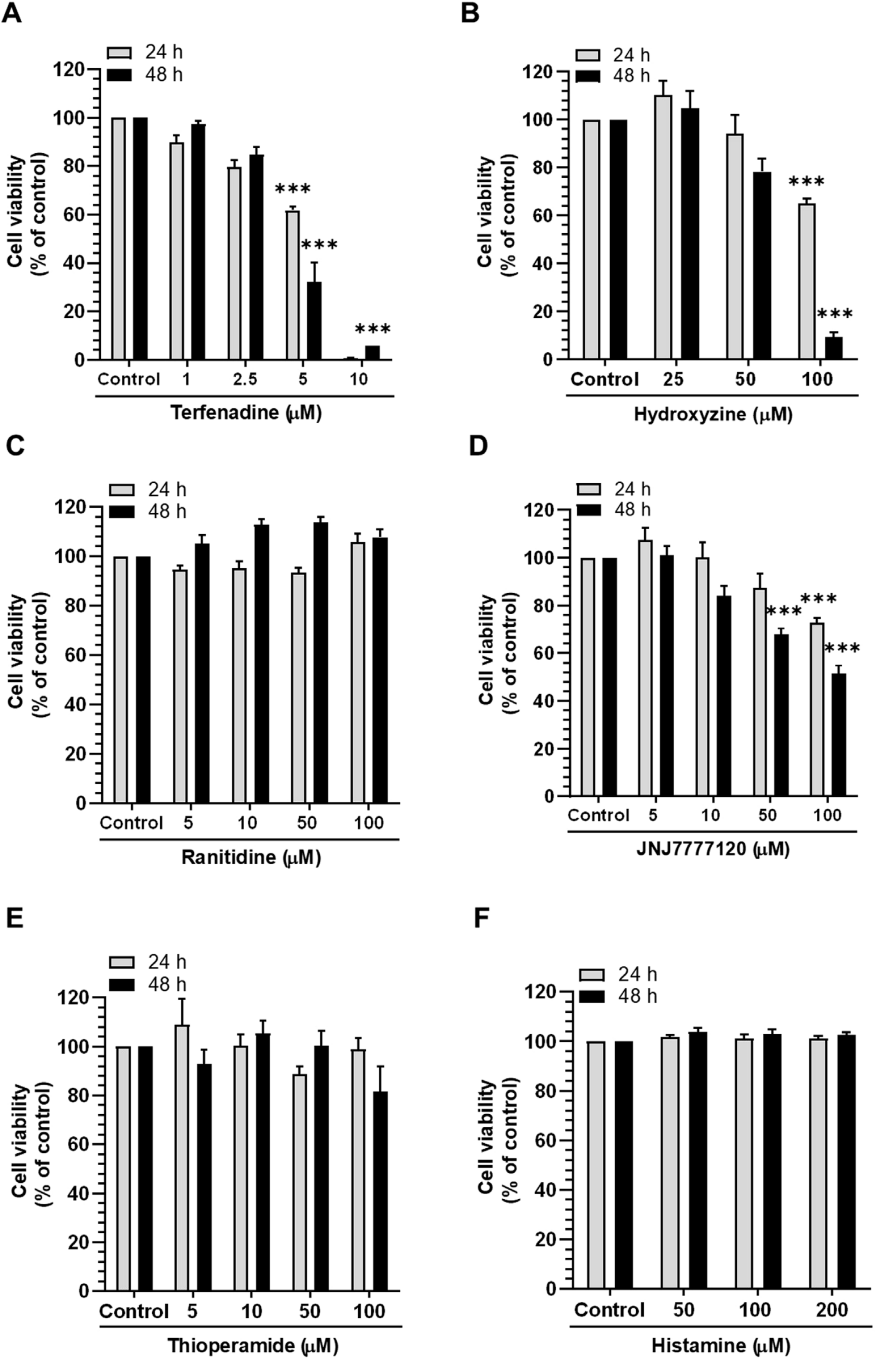
Effect of different histamine receptor antagonists and agonists on HCT116 cell viability. Cells were incubated with **(A)** terfenadine, **(B)** hydroxyzine, **(C)** ranitidine, **(D)** JNJ7777120, **(E)** thioperamide, and **(F)** histamine for 24 and 48 h. Cell viability was analyzed by the MTS assay. The results are presented as the mean ± SEM (*n* = 3). ***p < 0.001 *versus* control.

Accumulating evidence suggests that histamine receptors are overexpressed in CRC cells, which may be associated with disease progression (Nguyen and Cho, 2021); therefore, we inquired the effect of histamine receptor modulation on the growth of HCT116 cells using various antagonists and physiological agonists of histamine receptors. As presented in Figures 1A,B, the H1R antagonists terfenadine and hydroxyzine exert concentration- and time-dependent cytotoxic effects on HCT116 cells. However, treatment with ranitidine and thioperamide, H2R and H3R antagonists, respectively, did not significantly alter the viability of HCT116 cells (Figures 1C, E). Furthermore, treatment with the H4R antagonist JNJ7777120 at high concentrations (100 µM) was associated with significant cytotoxic effects (Figure 1D). We also assessed the effect of histamine, a physiological agonist of histamine receptors, on the viability of HCT116 cells. As a result, the growth of HCT116 cells was unaffected by histamine (Figure 1F). Overall, these outcomes provide strong proof for the crucial function of H1R or H4R in CRC progression, suggesting the possible use of antagonists of such receptors as potential anticancer agents for the management of CRC.

### 3.2 Terfenadine triggers apoptosis in HCT116 cells

**FIGURE 2:**
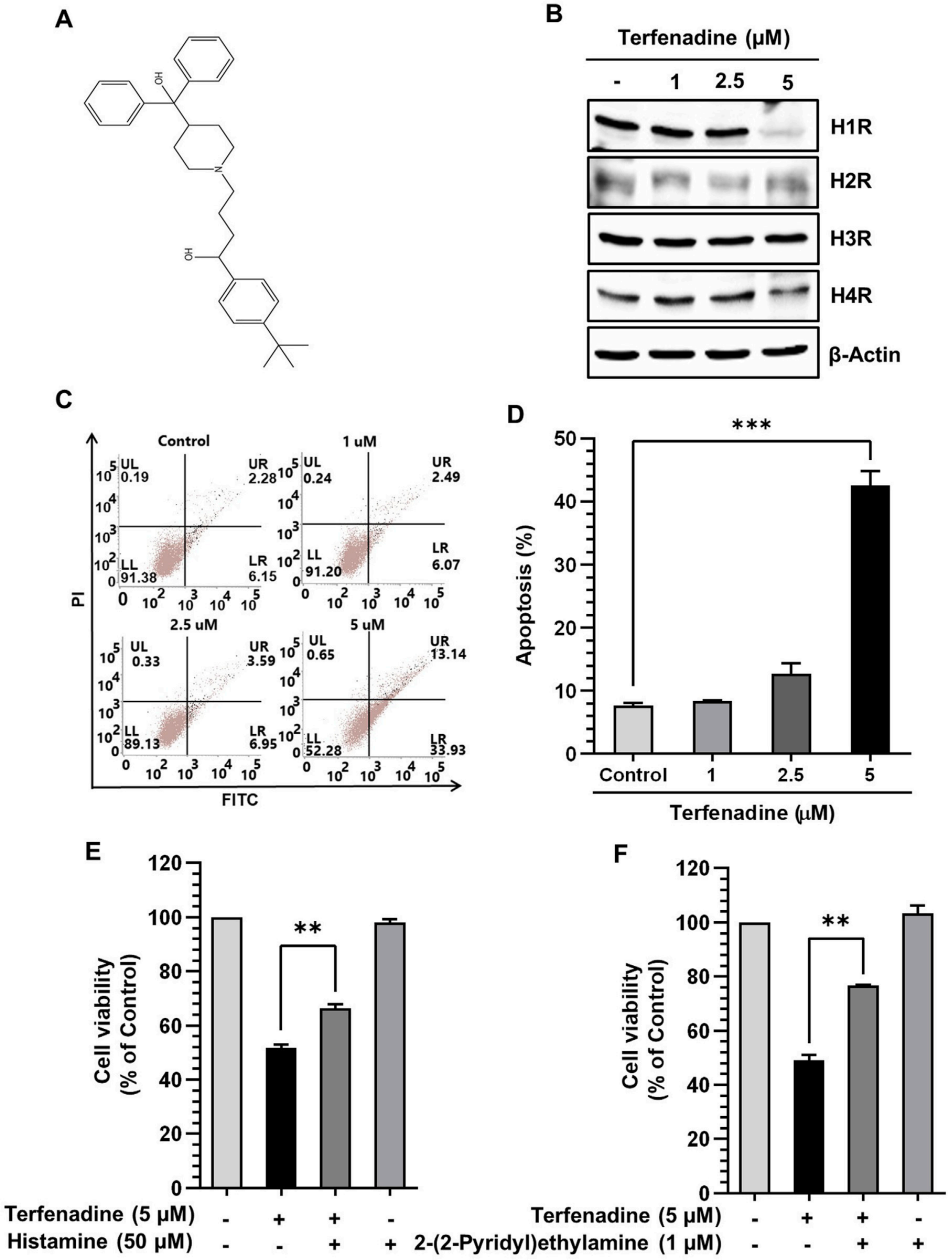
Effect of terfenadine on the apoptosis of HCT116 cells. **(A)** Chemical structure of terfenadine. **(B)** The effect of terfenadine on the expression of various histamine receptors was assessed by Western blot analysis. β-Actin represents loading control. **(C)** FACS analysis of the apoptotic effect of terfenadine employing Annexin V and PI staining. **(D)** Statistical data representing apoptosis percentage as the mean ± SEM (*n* = 3). ***p < 0.001 *versus* control. **(E, F)** Terfenadine was given with or without histamine or 2-(2-pyridyl)ethylamine for 24 h, and cell viability was analyzed. The data are plotted as the mean ± SEM (*n* = 3). **p < 0.01, and ***p < 0.001 *versus* terfenadine.

Figure 2A shows the chemical structure of terfenadine. We analyzed the effect of terfenadine on the expression levels of different histamine receptors. As shown in Figure 2B, terfenadine downregulated H1R expression in HCT116 cells. However, terfenadine did not significantly affect the expression levels of other histamine receptor subtypes, which highlights the selectivity of terfenadine toward H1R. Because terfenadine exerts potent cytotoxic effects on HCT116 cells (Figure 1A), we analyzed its effect on the provocation of apoptosis in HCT116 cells using Annexin V and PI staining. Terfenadine exerted a concentration-related surge in the number of cells undergoing apoptosis compared with untreated cells (Figures 2C,D), indicating that terfenadine-induced cell death is mediated through apoptosis. Pretreatment of cells with histamine and 2-(2-pyridyl)ethylamine, an H1R-selective agonist, partially reversed the cytotoxic effects of terfenadine (Figures 2E,F).

### 3.3 Terfenadine modulates apoptotic marker expression in HCT116 cells

**FIGURE 3:**
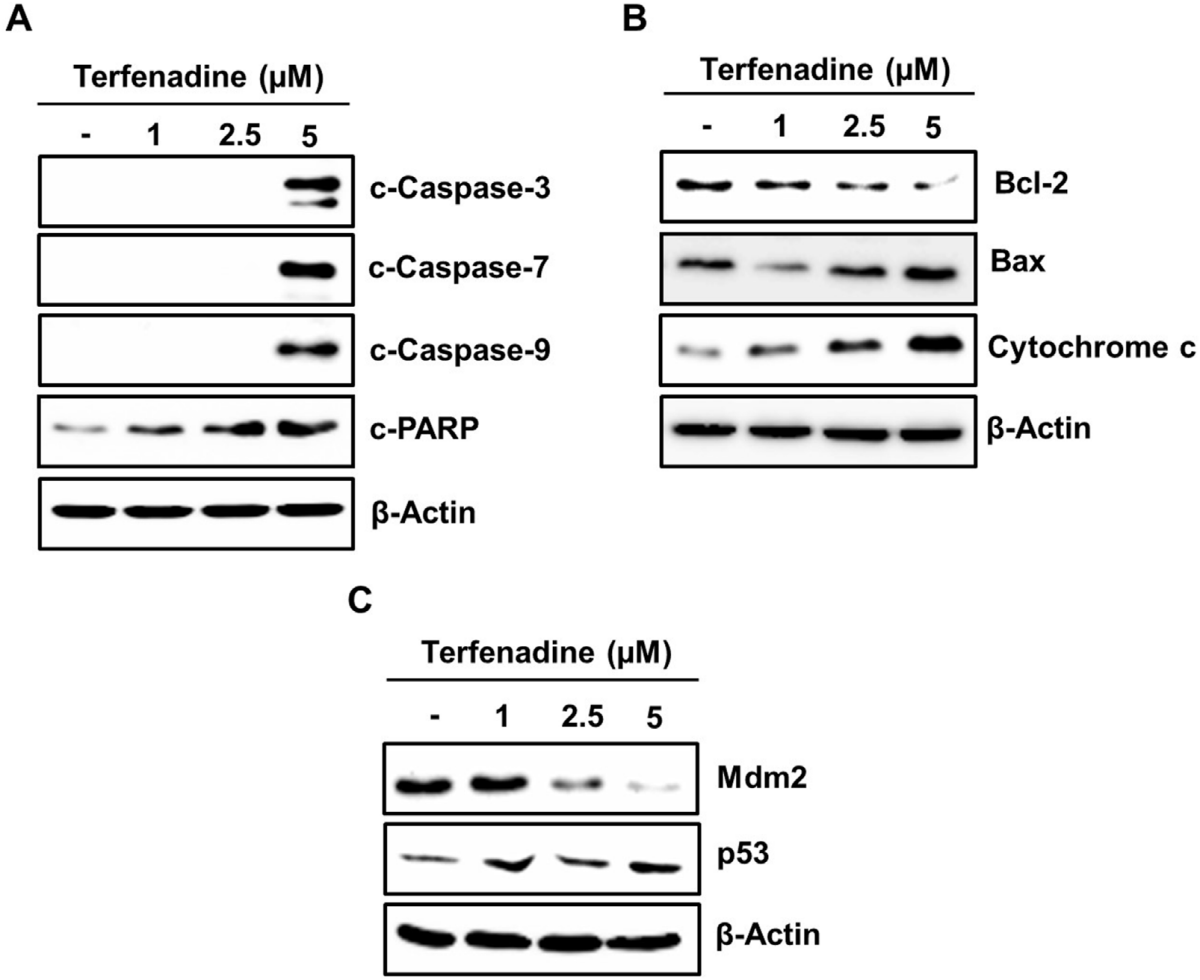
Impact of terfenadine on the modulation of apoptotic markers in HCT116 cells. **(A–C)** Cells were exposed to the specified strengths of terfenadine for 24 h. Western blot analysis to detect expression levels of **(A)** cleaved caspase-3, caspase-7, and caspase-9 and cleaved PARP, **(B)** Bcl-2, Bax, and cytochrome C, and **(C)** p53 and Mdm2. β-Actin represents a loading control.

Apoptosis involves stimulation of the caspase cascade through intrinsic or extrinsic pathways. Different stimuli triggering the modulation in the mitochondrial membrane potential cause the translocation of cytochrome c from the inner mitochondria to the cytoplasm. This phenomenon actuates procaspase-9, which in turn stimulates a sequence of caspase-3, caspase-6, and caspase-7 involved in PARP cleavage (Herr and Debatin, 2001). In this study, terfenadine treatment of cells demonstrated simultaneous actuation of caspase-3, caspase-7, and caspase-9 and PARP degradation (Figure 3A). This activation of the caspase cascade reveals that terfenadine-induced apoptosis involves an intrinsic pathway. The proper ratio of proapoptotic Bax and antiapoptotic Bcl-2 family proteins is a critical determinant of mitochondrial integrity. Therefore, we investigated the effect of terfenadine on these Bcl-2 family proteins. Interestingly, terfenadine significantly reduced Bcl-2 expression while upregulating Bax levels. Moreover, the level of cytoplasmic cytochrome c increased (Figure 3B). Whether terfenadine upregulated p53 levels was also investigated. In contrast, the constitutive levels of its counterpart, Mdm2, were concentration-dependently downregulated (Figure 3C). The amplification of the caspase cascade and elevated expression of p53 reveals that terfenadine-induced apoptosis involves an intrinsic pathway.

### 3.4 Terfenadine inhibits STAT3 phosphorylation and cyclin and survivin expression in HCT116 cells

**FIGURE 4:**
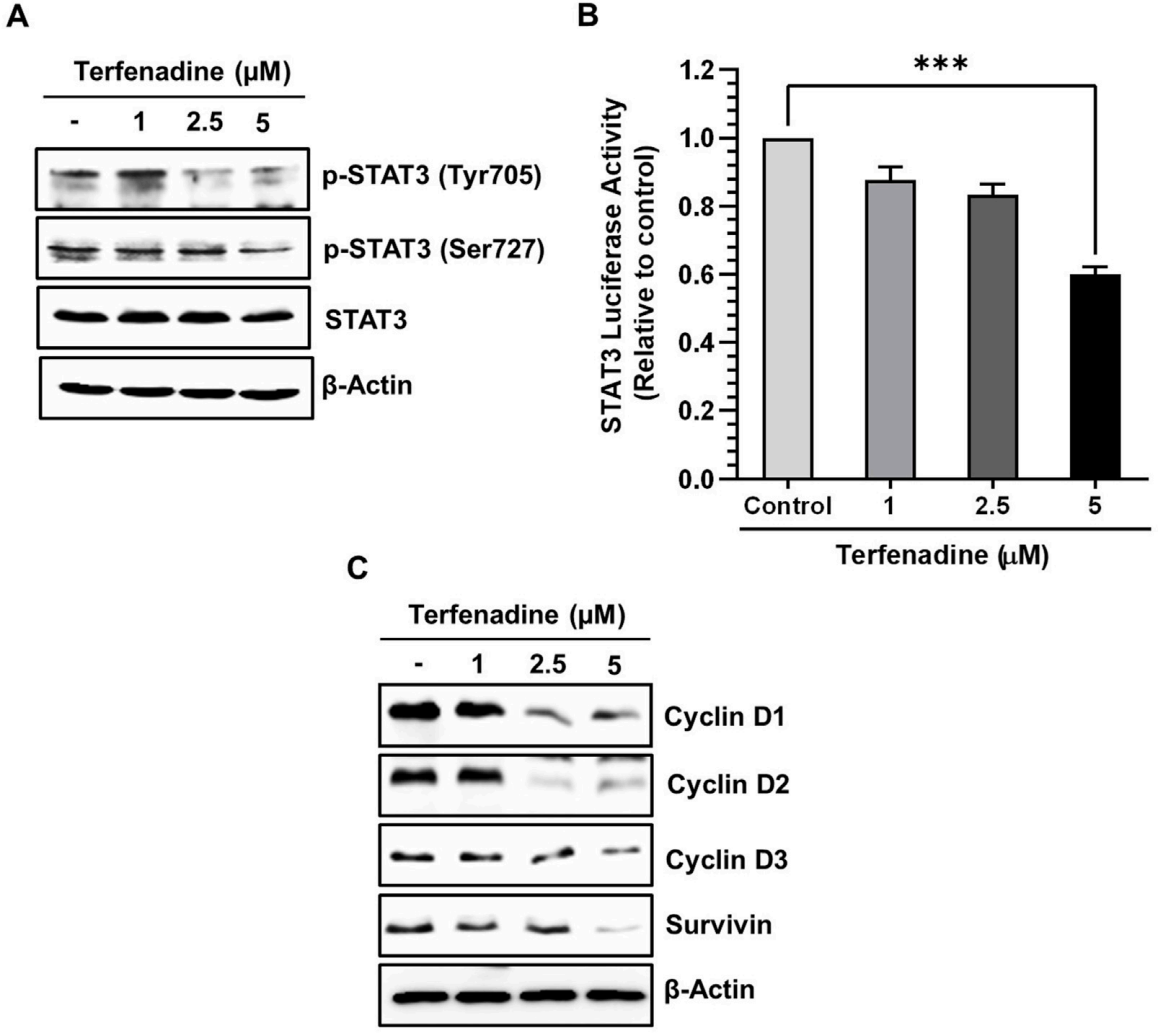
Terfenadine impedes the actuation of STAT3 and its transcriptional activity, contributing to diminished levels of its gene products. **(A)** Varying concentrations of terfenadine were applied to the cells for 24 h. Western blot analysis was carried out to quantify the levels of p-STAT3 (Tyr705) and p-STAT3 (Ser727). **(B)** STAT3 and Renilla luciferase reporter plasmids were transfected into the cells, which were then exposed to varying strengths of terfenadine for 24 h. Luciferase assay was examined employing a dual luciferase reporter system. Results are plotted as the mean ± SEM (*n* = 3). ***p < 0.001 *versus* control. **(C)** Western blot analysis to estimate levels of cyclin D1, cyclin D2, cyclin D3, and survivin upon exposure of cells with terfenadine for 24 h.

STAT3 is aberrantly stimulated in different cancers, including colorectal carcinoma (Dobi et al., 2013). The aberrant activation of STAT3 positively regulates the transcription of several cell survival genes, thus acting as a critical mediator of carcinogenesis (Aggarwal et al., 2009). We investigated the effect of terfenadine on STAT3 phosphorylation in HCT116 cells. Terfenadine attenuated STAT3 activation at tyrosine705 and serine727 residues (Figure 4A). Similarly, the transcriptional activity of STAT3 in HCT116 cells substantially decreased (Figure 4B). In addition, expression levels of survivin, cyclin D1, cyclin D2, and cyclin D3 were markedly suppressed during the incubation of cells with terfenadine (Figure 4C).

### 3.5 Terfenadine-induced STAT3 inactivation is associated with the downregulation of MEK/ERK and JAK2 phosphorylation in HCT116 cells

**FIGURE 5:**
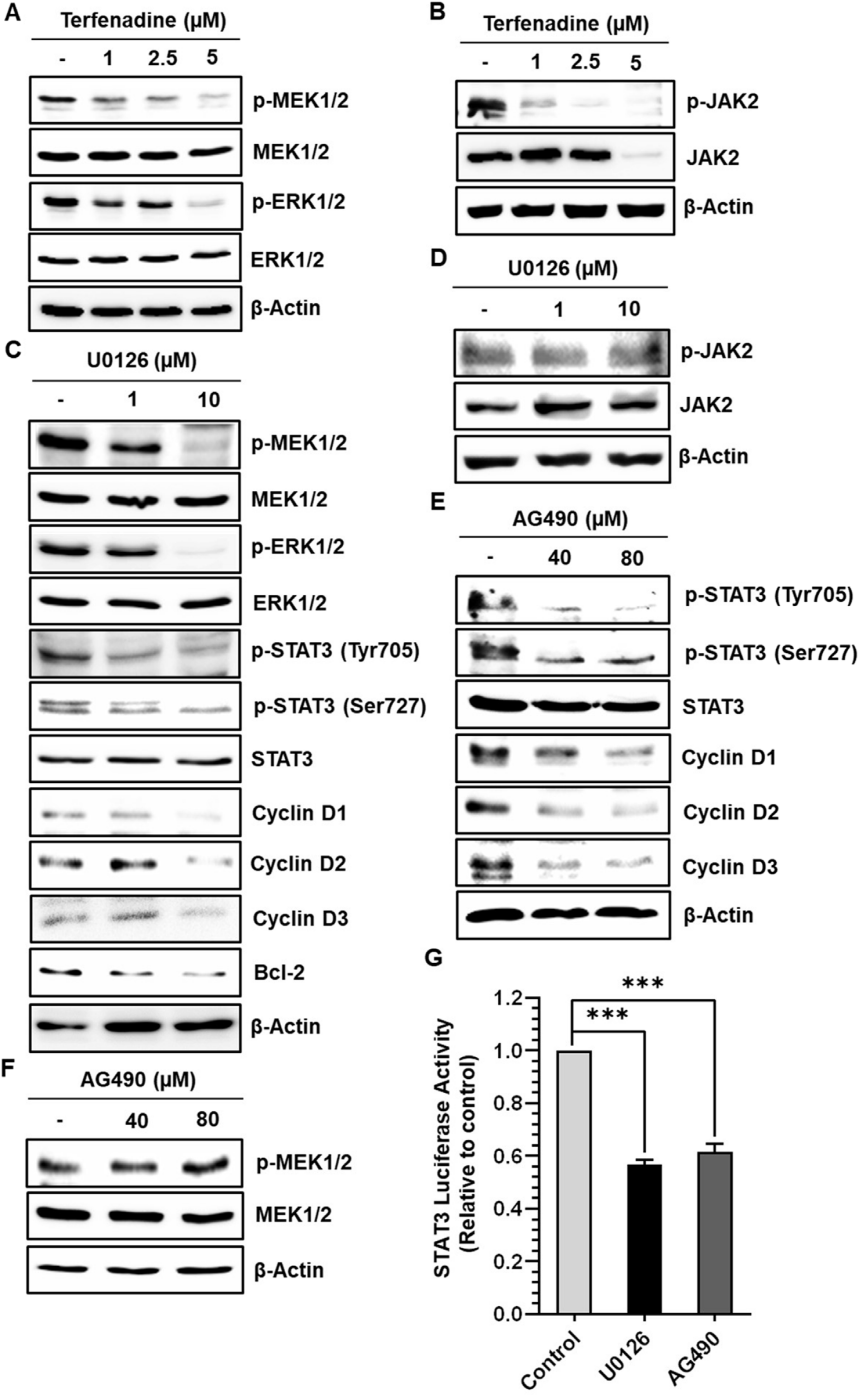
Terfenadine inhibits STAT3 activation and its downstream targets by suppressing MEK1/2 and JAK2 phosphorylation in HCT116 cells. **(A, B)** Cells were incubated with the specified strengths of terfenadine for 24 h **(A)** p-MEK1/2 and p-ERK1/2 levels were assessed by immunoblot analysis. **(B)** Western blot analysis to check the levels of p-JAK2. **(C, D)** Cells were exposed to the specified concentration of U0126 for 24 h. **(C)** Western blot analysis was employed to determine the levels of p-MEK1/2, p-ERK1/2, p-STAT3 (Tyr705), p-STAT3 (Ser727), cyclins, and Bcl-2. **(D)** The protein level of p-JAK2 was assessed by Western blot analysis. **(E, F)** Cells were exposed to the specified strength of AG490 for 24 h. **(E)** The protein levels of p-STAT3 (Tyr705), p-STAT3 (Ser727), and different cyclins were determined by Western blot analysis. **(F)** The protein level of p-MEK1/2 was assessed by Western blot analysis. **(G)** Cells were transfected with two types of plasmids, one containing a STAT3 luciferase reporter and the other containing a Renilla luciferase reporter, and then treated with U0126 (10 µM) or AG490 (80 µM) for 24 h after transfection. Luciferase activity was quantified using dual luciferase reporter assay. The results are expressed as the mean ± SEM (*n* = 3). ***p < 0.001 *versus* control.

**FIGURE 6:**
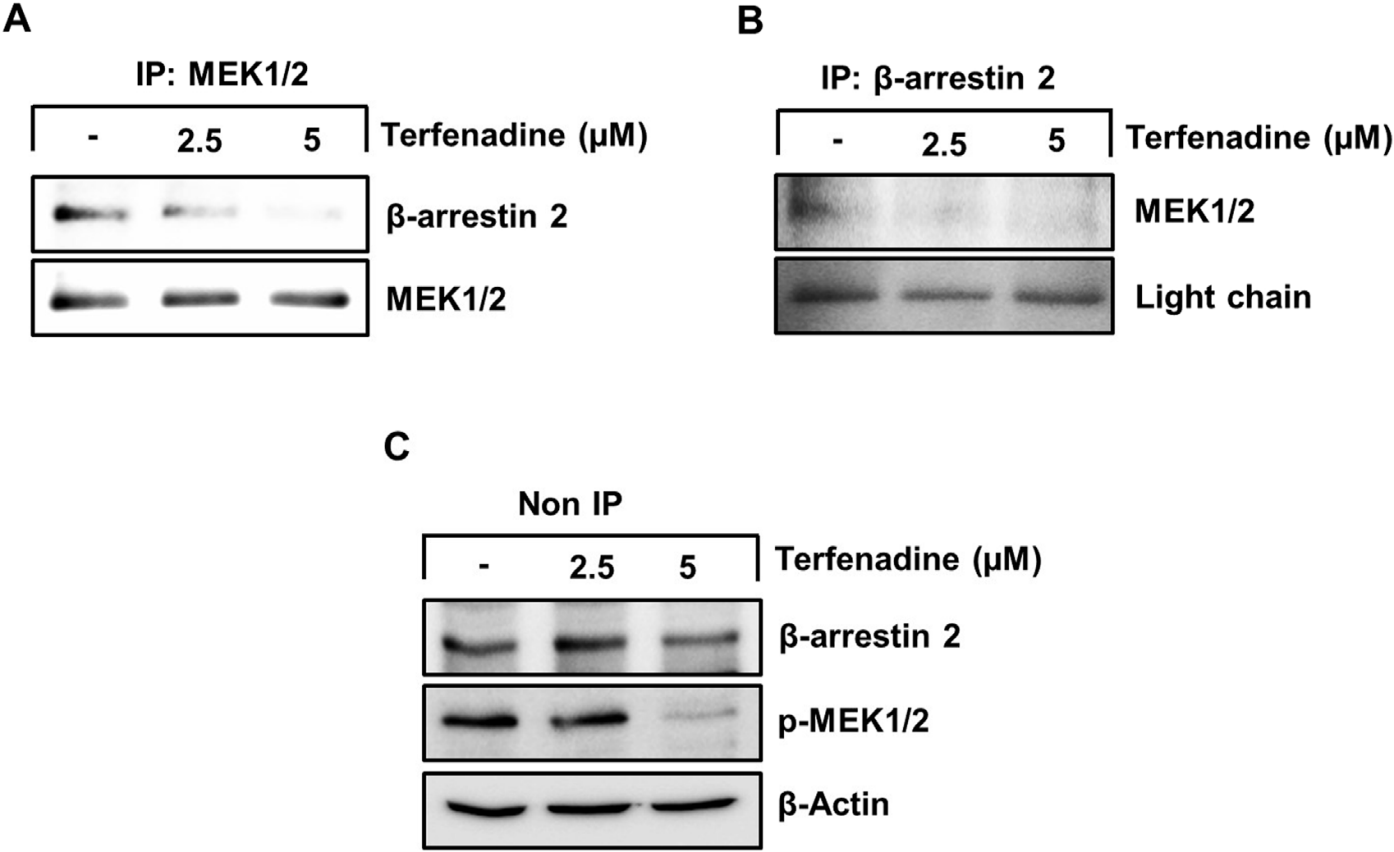
Terfenadine inhibits the complex formation of β-arrestin 2 and MEK in HCT116 cells. **(A–C)** Cells were exposed to varying strengths of terfenadine for 24 h. The levels of β-arrestin 2 and MEK1/2 were assessed by immunoprecipitation assay **(A, B)** and immunoblotting **(C)**.

STAT3 activation is regulated by various upstream kinases, including JAK2 (Slattery et al., 2013) and ERK (Aggarwal et al., 2009). The current research shows that terfenadine downregulates the phosphorylation of MEK, ERK (Figure 5A), and JAK2 (Figure 5B) in HCT116 cells. Surprisingly, total JAK2 expression was also suppressed by terfenadine. As depicted in Figure 5C, U0126, a pharmacological inhibitor of MEK, markedly suppressed MEK and ERK phosphorylation and subsequent STAT3 inactivation. In addition, U0126 treatment decreased the levels of STAT3-targeted proteins, such as cyclins D1, D2, and D3 and Bcl-2 in HCT116 cells (Figure 5C). However, U0126 did not affect the phosphorylation of JAK2 (Figure 5D). Similarly, STAT3 phosphorylation at tyrosine705 and serine727 residues and expression levels of cyclins in HCT116 cells upon treatment with AG490, which is a JAK2 inhibitor, were markedly downregulated (Figure 5E). In addition, the levels of p-MEK1/2 remained unchanged upon AG490 treatment (Figure 5F). Moreover, the *STAT3* gene reporter activity was significantly inhibited by U0126 and AG490 (Figure 5G). Collectively, these findings indicate that MEK/ERK and JAK2 appear to be mutually independent upstream kinases of STAT3 and that their inhibition downregulates the transcriptional activity of STAT3 in HCT116 cells. Moreover, we outlined the impact of terfenadine on the G protein-independent pathway in HCT116 cells. The result shows that terfenadine suppressed the interaction between MEK and β-arrestin 2 as determined by immunoprecipitation using the MEK antibody (Figure 6A). This decrease in complex formation was further confirmed by reverse immunoprecipitation using β-arrestin 2 antibody (Figure 6B). As shown in Figure 6C, the immunoblot analysis demonstrates that MEK phosphorylation was suppressed by terfenadine, whereas the level of β-arrestin 2 remained constant. This projects that β-arrestin 2 function as a scaffold for MEK, which causes subsequent MEK phosphorylation through the G protein-independent pathway.

### 3.6 Inactivation of PKC substrates by terfenadine downregulates MEK/ERK signaling in HCT116 cells

**FIGURE 7:**
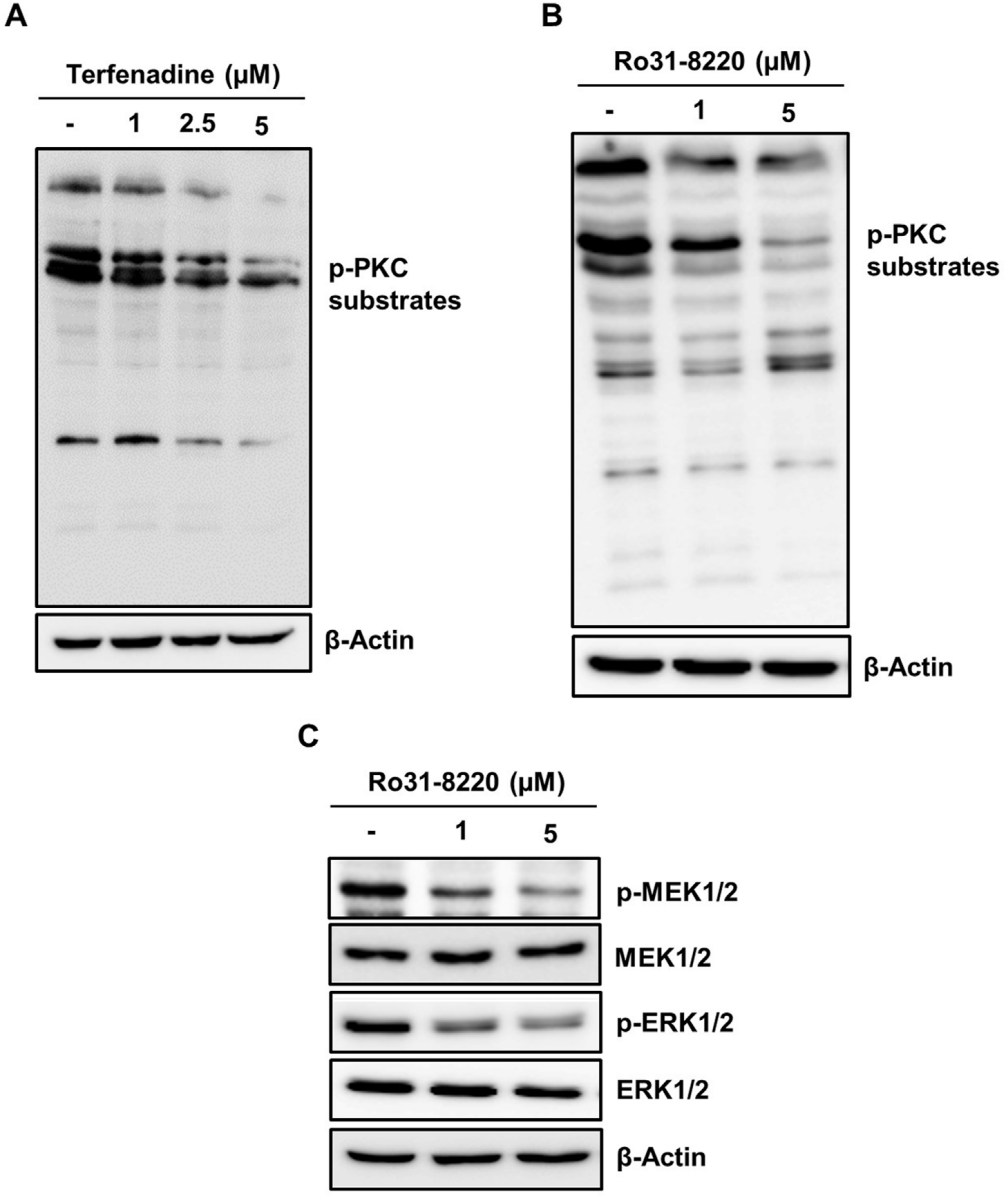
Inactivation of PKC substrates by terfenadine downregulates MEK/ERK signaling in HCT116 cells. **(A)** Cells were exposed to terfenadine for 24 h at the specified strengths. The impact of terfenadine on the phosphorylation of PKC substrates was evaluated by Western blot analysis. **(B, C)** Cells were exposed to Ro31-8220 for 24 h at the specified strengths. **(B)** Expression of p-PKC substrates was determined by immunoblot analysis. **(C)** Phosphorylation of MEK1/2 and ERK1/2 was assessed by Western blot analysis.

PKC has been reported to exert a pivotal function in carcinogenesis or cancer progression through the activation of various downstream pathways such as MEK/ERK signaling (Matsubara et al., 2005; Isakov, 2018); therefore, we inquired the impact of terfenadine on the activation of PKC substrates. Terfenadine suppressed the phosphorylation of PKC substrates (Figure 7A). Similarly, Ro31-8220, a pan-PKC inhibitor, downregulated the phosphorylation of PKC substrates in HCT116 cells (Figure 7B). In addition, the effect of Ro31-8220 on the modulation of MEK/ERK signaling was studied to elucidate the relationship between PKC and MEK/ERK signaling. Interestingly, Ro31-8220 markedly suppressed the phosphorylation of MEK and ERK (Figure 7C), suggesting that PKC inhibition abrogates MEK/ERK activation.

### 3.7 Terfenadine abrogates the growth of HCT116 tumor xenografts *in vivo*

**FIGURE 8:**
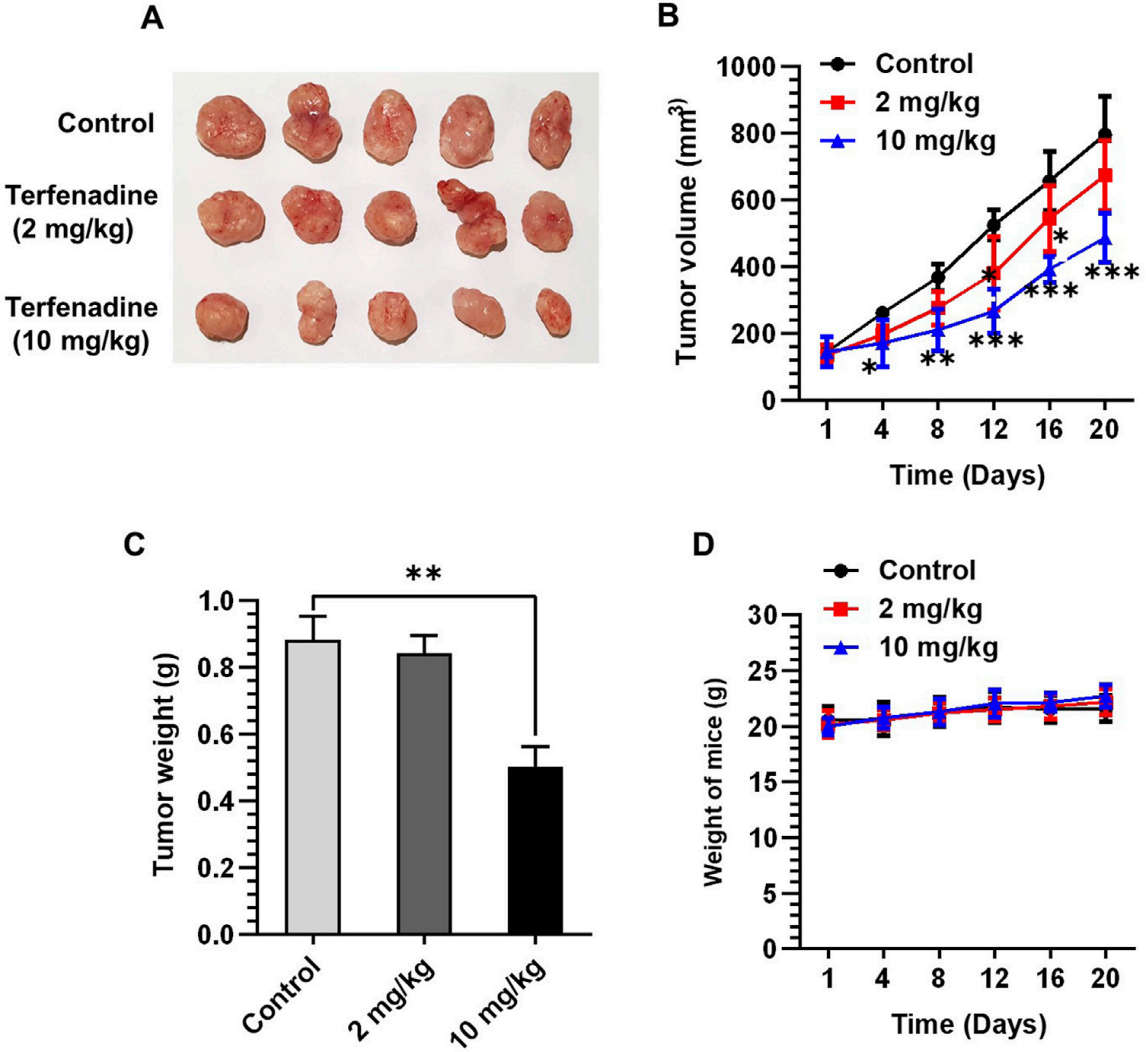
Terfenadine suppresses the growth of HCT116 tumor xenografts. **(A–D)** HCT116 cells were mixed with matrigel in equal proportion and inoculated into the right flank of BALB/c nude mice to create tumor xenografts. The mice were randomly split into three groups when the tumor volume measured around 100–150 mm^3^: control (corn oil) and terfenadine groups (2 and 10 mg/kg). Terfenadine was administered daily through intraperitoneal injection. **(A)** Excised tumor from mice after terfenadine injection for 20 days. **(B)** Plot of the tumor volume over 20 days. **(C)** Tumor weight after excision from mice was measured. **(D)** Mice weight was recorded twice per week for up to 20 days. Values are plotted as the mean ± SEM (*n* = 5). **p < 0.01, ***p < 0.001 *versus* control.

Given the marked tumor suppressive effect of terfenadine *in vitro*, we attempted to examine its anticancer effect *in vivo* by generating an HCT116 xenograft model in BALB/c nude mice. After successful induction of tumor xenografts, mice were administered terfenadine intraperitoneally daily at two doses (2 and 10 mg/kg). After 1 week of daily administration of terfenadine, the reduction in tumor volume was clearly noticeable. As shown in Figure 8A, terfenadine injection (10 mg/kg) markedly impeded the growth of the HCT116 tumor xenograft. Moreover, terfenadine significantly reduced the tumor volume at different time points compared with the corn oil group (Figure 8B). In addition, the weight of the tumor excised from the terfenadine group was comparatively lower than the control (Figure 8C). The weight of the mice was also monitored throughout the experiment. Interestingly, no significant deviations in the body weights of the mice were found in all three groups, indicating that the terfenadine dosage was safe and nontoxic to the mice (Figure 8D). Overall, these results suggest that terfenadine exerts a prominent antitumorigenic effect *in vivo*, which agrees well with its *in vitro* growth inhibitory effect.

## 4 Discussion

Despite novel advancements in anticancer drug development, successful treatment of CRC remains an immense challenge. CRC is the third most prevalent cancer and is considered the second dominant origin of cancer-related deaths. CRC accounts for 10.2% globally, which is expected to increase in the near future (Biller and Schrag, 2021; Sung et al., 2021). The potential implications of this projection have garnered worldwide attention for the development of effective therapies for CRC, emphasizing the pathophysiological factors contributing to the disease.

Histamine is considered a dominant factor in the etiology and advancement of CRC (Massari et al., 2020). CRC tissues exhibit high levels of histamine in contrast to healthy tissues (Chanda and Ganguly, 1987; Reynolds et al., 1997). A clinical study revealed that patients with malignant solid tumors had significantly higher blood histamine levels, which were normalized after surgical excision of the cancer tissues (Moriarty et al., 1988). Moreover, the function of the histidine decarboxylase enzyme, which is involved in histamine synthesis, is reported to be two-fold higher in CRC specimens than in healthy tissues (Garcia-Caballero et al., 1988; Moriarty et al., 1988). Exogenous administration of histamine accelerated the growth of *in vivo* tumor xenografts in mice, which supports the positive association between histamine and CRC (Tomita and Okabe, 2005). In contrast, numerous studies have suggested an anti-tumorigenic function of histamine in cancer (Parihar et al., 2013; Gao et al., 2017). These controversial role of histamine in carcinogenesis are assumed to be dependent on the variable interplay between histamine and histamine receptors and interactions with other cellular components of the tumor microenvironment (Massari et al., 2020). Thus, the biphasic role of histamine and its differential interactions with histamine receptors prompted us to screen the effect of various classes of histamine receptor antagonists on the viability of CRC HCT116 cells. We investigated whether terfenadine and hydroxyzine, H1R antagonists, exert concentration- and time-dependent cytotoxic effects on HCT116 cells. Moreover, JNJ7777120, an H4R antagonist, decreased cell growth in a concentration- and time-dependent pattern. However, the intensity of cytotoxicity with JNJ7777120 was lower than that with terfenadine and hydroxyzine (Figures 1A,B,D). In contrast, neither H2R and H3R antagonists nor histamine elicited antiproliferative effects on HCT116 cells. Although histamine acts as a physiological agonist for all four types of histamine receptors, the signaling mechanisms elicited by histamine upon interaction with individual histamine receptors are different. The role of individual histamine receptors in CRC is contradictory and context-dependent. While activation of H1R is suggested to drive CRC progression, stimulation of other receptors may exhibit opposite effects (Massari et al., 2020; Nguyen and Cho, 2021). Zhongcheng et al. reported distinct roles of H1R and H2R in colonic tumorigenesis. According to the study, H1R signaling stimulates and H2R signaling suppresses the proliferation of CRC cells. Interestingly, H2R activation counteracted the H1R signaling (Shi et al., 2019). Due to non-specific affinity to histamine receptors and differential activity of histamine, it is postulated that the overall effect of histamine on viability of HCT116 cells could be neutralized which could be the reason for no significant alteration of viability when the cells were stimulated with histamine. These findings are in good correlation with the overexpression of H1R in CRC, which represents a positive connection between H1R and the progression of CRC (Nguyen and Cho, 2021). In addition, we investigated the cytotoxic effect of terfenadine in Caki-1, SK-MEL-28, and U87MG cells and found that terfenadine suppressed the viability of these cancer cells too which suggests broad-spectrum anticancer effects of terfenadine (Supplementary Figure S1). However, it requires extensive research work to validate its efficacy in other cancer types. The evidence of the variable effects of histamine receptor antagonists on HCT116 cells drove us to further investigate the anticancer properties of terfenadine and decipher its underlying molecular mechanisms.

The clinical use of terfenadine for the treatment of allergies was associated with cardiotoxicity which caused its withdrawal from the market. This raises concern regarding the clinical translation of the drug as anticancer therapy, however, at that time the medicine was available in conventional dosage forms. Owing to the presence of H1R in the heart, it was more likely to occur such adverse effects due to the direct antagonistic effect of terfenadine (MacConnell and Stanners, 1991). However, various advancements in drug delivery technologies such as nano-based delivery approaches along with active targeting opened the door to achieving selective drug delivery to cancer cells thus minimizing the adverse effects and sparing healthy cells from the toxic effects of the drug (Fan et al., 2023).

Histamine-mediated activation of H1R is associated with the upregulation of H1R expression via an autoregulatory loop involving PKC isoforms (Mizuguchi et al., 2011; Hattori et al., 2013; Mizuguchi et al., 2021). Therefore, we investigated how terfenadine affects the expression of various histamine receptors, i.e., H1R, H2R, H3R, and H4R. Intriguely, terfenadine selectively downregulated the expression of H1R without a significant impact on the level of other types of histamine receptors, which confirms its specificity toward H1R (Wang et al., 2014). Consistent with this, it was found that treatment of U-373 cells with H1R antagonist suppressed the expression of H1R level via PKCα isoform (Mizuguchi et al., 2021). Moreover, another study also demonstrated downregulation of H1R expression upon inactivation of H1R signaling using quercetin through suppression of protein kinase C-δ/extracellular signal-regulated kinase/poly (ADP-ribose) polymerase-1 signaling pathway in HeLa cells (Hattori et al., 2013). In addition, studies report upregulation of the expression of H1R as a result of histamine-mediated stimulation (Mizuguchi et al., 2011; Mizuguchi et al., 2021). Based on these findings, it seems plausible to propose that inhibition of H1R signaling using its specific antagonists is likely to cause downregulation of H1R via PKC-dependent signaling. However, extensive studies are required to unveil the mechanism of H1R downregulation by terfenadine in HCT116 cells. As demonstrated in Figure 1A, depending on the cytotoxic effect of terfenadine in HCT116 cells, we then attempted to elucidate whether terfenadine-induced cell death proceeded through the apoptotic mechanism. Terfenadine administration triggered concentration-dependent apoptosis in HCT116 cells, demonstrating a good correlation with its cytotoxic effects. These outcomes are congruent with the apoptotic effects of terfenadine unveiled by several previous studies (Liu et al., 2003; Nicolau-Galmés et al., 2011). Moreover, pretreatment with histamine or 2-(2-pyridyl)ethylamine, agonists of H1R, partially restored cell viability (Figures 2E,F). This suggests that terfenadine-induced cell death occurs partially through H1R inhibition. Previous studies from our laboratory demonstrated that aberrant reactive oxygen species (ROS) production can enhance apoptosis in CRC cells (Chae et al., 2014; Park et al., 2014; Kim et al., 2016). Herein, ROS production in HCT116 cells was observed upon terfenadine treatment (Supplementary Figure S2). Thus, we speculate that terfenadine-induced ROS production may be a partial contributor to the cytotoxic effects of terfenadine, which requires further research. Several other studies have also supported the notion that terfenadine triggers H1R-mediated and independent pathways as cell death mechanisms (Jangi et al., 2008; Wang et al., 2014).

Caspase-9 actuation serves as a major determinant of intrinsic apoptosis (Goldar et al., 2015). This study depicted that terfenadine provoked the stimulation of the caspase cascade, leading to the disruption of PARP functionality, suggesting that an intrinsic mechanism is involved in terfenadine-triggered apoptosis in HCT116 cells. Alterations in the Bax/Bcl-2 ratio prompts the discharge of cytochrome c from the mitochondria, which in turn triggers the caspase cascade (Elmore, 2007; Park et al., 2016; An et al., 2022). In this study, terfenadine upregulated the constitutive levels of the proapoptotic protein Bax, whereas the level of Bcl-2 was reduced. Thus, this change in the homeostasis of proapoptotic and antiapoptotic proteins may be associated with terfenadine-induced cell death. p53 directs the transcriptional activation of Bcl-2 family proteins, including Bax (Miyashita et al., 1994). Terfenadine upregulated p53 levels, which may be responsible for the shift in the Bax/Bcl2 ratio and activation of the caspase cascade. Moreover, terfenadine-induced downregulation of Mdm2, which is upstream of p53, advocates the crucial function of p53 in cell death.

STAT3 exhibits an oncogenic nature and is constitutively activated in different cancers, including CRC (Dobi et al., 2013; Khatoon et al., 2023). It participates in the transcriptional modulation of various genes linked to cell cycle progression and survival. Phosphorylation leads to the dimerization and nuclear dislocation of STAT3, which is closely linked to its transcriptional activity (Dobi et al., 2013; Raut et al., 2021). Aberrant stimulation of STAT3 facilitates cancer cell growth, whereas hindrance of STAT3 signaling triggers apoptosis and cell cycle suppression in colon cancer cells (Corvinus et al., 2005; Lin et al., 2005; Chae et al., 2014; Kim et al., 2016). This led us to examine the effect of terfenadine on STAT3 phosphorylation in HCT116 cells. Interestingly, terfenadine suppressed the phosphorylation of STAT3 at tyrosine705 and serine727 residues along with the downregulation of the STAT3 reporter gene assay (Figure 4). Moreover, terfenadine diminished the expression levels of STAT3-regulated genes, including cyclins and survivin, which is congruent with the results of earlier studies reporting terfenadine-induced suppression of cyclins in other categories of CRC cells (Liu et al., 2003). These results support the oncogenic nature of STAT3 and its involvement in HCT116 cell proliferation.

STAT3 activation is regulated by several upstream kinases, including JAK2 (Slattery et al., 2013; Kim et al., 2017) and MEK/ERK (Aggarwal et al., 2009). This study showed that terfenadine decreased the activation of JAK2 and MEK/ERK. Surprisingly, the total level of JAK2 was also suppressed; therefore, whether the attenuation of phosphorylation of JAK2 by terfenadine was caused by a decrease in the total JAK2 levels is difficult to rule out. JAK2 mutation in leukemia cells may result in the proteasomal degradation of JAK2, consequently leading to a decline JAK2 levels (Marubayashi et al., 2010). However, CRC cells do not carry the JAK2 mutation (Herreros-Villanueva et al., 2010). Heat shock protein 90 (HSP90), a chaperone protein, maintains the stability of several client proteins, including JAK2 (Park et al., 2015). ROS production can facilitate HSP90 cleavage and inactivation, consequently leading to JAK2 degradation (Chae et al., 2014; Park et al., 2015). Indeed, terfenadine increased ROS production in a concentration- and time-dependent pattern (Supplementary Figure S2). Therefore, the terfenadine-induced ROS production may be involved in JAK2 degradation. However, further research is required to unveil the mechanism of total JAK2 reduction by terfenadine in HCT116 cells. Moreover, to investigate the interplay of JAK2 and MEK/ERK in STAT3 activation in HCT116 cells, specific pharmacological inhibitors were used. Remarkably, AG490 and U0126 inhibited STAT3 phosphorylation and its transcriptional functions (Figures 5C,E). Moreover, the reduction in the STAT3 reporter gene activity by the JAK2 and MEK inhibitors provides supporting evidence that these kinases serve as the upstream of STAT3 in HCT116 cells. Moreover, the JAK2 inhibitor did not affect MEK phosphorylation and *vice versa* (Figures 5D,F), which highlights the involvement of two independent signaling mechanisms affected by terfenadine in HCT116 cells.

H1R can activate the MAPK pathway by recruiting β-arrestins or downstream signaling cascade of G proteins, including PKC, which can activate MEK and ERK (Matsubara et al., 2005; Jain et al., 2016). Because the action of terfenadine is partially H1R-dependent, we analyzed the effect of terfenadine on the phosphorylation of PKC substrates. In this study, terfenadine decreased the phosphorylation of PKC substrates. Furthermore, Ro31-8220, a pan-PKC inhibitor, downregulated the phosphorylation of PKC substrates and MEK/ERK in HCT116 cells, indicating that terfenadine decreases STAT3 activity through the PKC/MEK/ERK axis. The presented results align with earlier findings of the suppression of MEK/ERK signaling downstream of PKC using other H1R antagonists in human epidermal keratinocytes (Matsubara et al., 2005; Aziz et al., 2010). However, we did not investigate the involvement of specific PKC isoforms in this signaling, which warrants further research. Moreover, terfenadine decreased the recruitment and complex formation of β-arrestin 2 with MEK (Figures 6A–C). These findings indicate that terfenadine simultaneously downregulates H1R signaling through G protein-dependent and G protein-independent mechanisms in HCT116 cells.

We extended the scope of this research through *in vivo* studies using an HCT116 xenograft model to validate the cytotoxic effects of terfenadine. As illustrated in Figures 8A–C, tumor growth was markedly repressed by terfenadine administration to mice. The outcomes of the *in vivo* study correlate with the *in vitro* cytotoxic effects of terfenadine, which are in good agreement with the results of other studies where terfenadine administration retarded the growth of MDA-MB-231 cells (Fernández-Nogueira et al., 2018) and hepatocellular carcinoma (Zhao et al., 2020).

**FIGURE 9:**
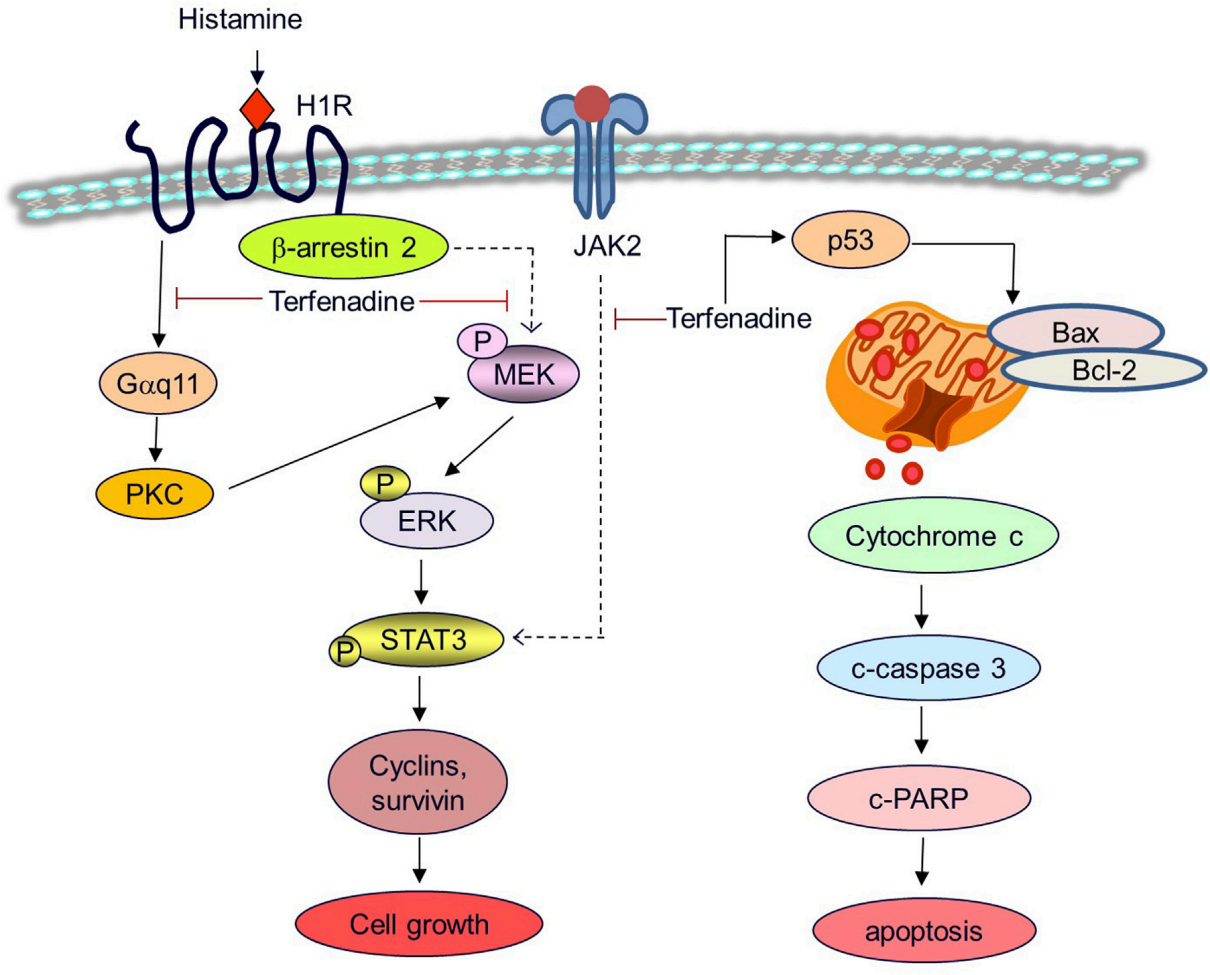
A proposed mechanistic model for the anticancer effects of terfenadine in HCT116 cells. Terfenadine triggers apoptosis in HCT116 cells via the intrinsic pathway. Terfenadine modulates the balance of Bax and Bcl-2 which causes the release of cytochrome c into the cytoplasm consequently activating caspase cascade and degradation of PARP. Moreover, terfenadine suppresses G protein-mediated and β-arrestin 2-dependent activation of MEK and ERK. In addition, it downregulates JAK2 phosphorylation. Inactivation of MEK and JAK2 leads to abrogation of STAT3 phosphorylation and expression of STAT3-dependent genes including cyclins and survivin causing inhibition of the growth of colorectal cancer HCT116 cells.

This study illustrates that terfenadine provokes apoptosis and retards the growth of HCT116 cells. Terfenadine activates the caspase cascade through the intrinsic apoptotic pathway. Moreover, terfenadine inhibited the activation of PKC substrates and MEK/β-arrestin 2 complex formation as well as the phosphorylation of JAK2, which ultimately downregulated STAT3 activation and its transcriptional activity, resulting in reduced expression of cyclins and survivin. On the basis of the results of this study, we have outlined the proposed mechanism of action for the anticancer effect of terfenadine in Figure 9. The antitumor effects of terfenadine in the HCT116 tumor xenograft model strongly correlate with the *in vitro* tumor suppression effects. Overall, the outcomes of this study elucidate that terfenadine is a potential anticancer agent for managing CRC.

## Data Availability

The raw data supporting the conclusion of this article will be made available by the authors, without undue reservation.
